# Management of Refractory Malignancy-Associated Hemophagocytic Lymphohistiocytosis in Adolescent Patients: A Case Series of Novel Therapeutics and Treatment Challenges

**DOI:** 10.3390/hematolrep17030028

**Published:** 2025-05-20

**Authors:** Meha Krishnareddigari, Kenny Vo, Arun Panigrahi

**Affiliations:** 1 UCLA Health, Los Angeles, CA 90025, USA; meha.krishna1@gmail.com (M.K.); khvo@mednet.ucla.edu (K.V.); 2 UC Davis Health, Sacramento, CA 95817, USA

**Keywords:** HLH, lymphohistocytosis, malignancy

## Abstract

Background: Hemophagocytic lymphohistiocytosis (HLH) is a potentially fatal syndrome of immune dysregulation with primary (genetic) and secondary (acquired) forms, including malignancy-associated HLH (m-HLH). The condition often presents significant diagnostic and therapeutic challenges due to overlapping symptoms with underlying malignancies and the absence of standardized guidelines for refractory cases. The established standard of care is dexamethasone and etoposide, but no guidelines exist for refractory HLH or cases triggered by malignancy. Case presentations: This case series describes three adolescent patients with m-HLH, focusing on complexities in diagnosis, treatment regimens, and toxicity management. While dexamethasone and etoposide remain a standard of care, their efficacy in refractory cases is limited. We highlight the novel use of targeted therapies, including emapalumab, an interferon-gamma inhibitor, and ruxolitinib, a JAK1/2 inhibitor, which showed potential in modulating immune hyperactivation. Conclusions: Our findings emphasize the need for individualized treatment approaches in adolescent m-HLH and importance of further research to establish evidence-based therapeutic guidelines for refractory cases.

## 1. Introduction

Hemophagocytic lymphohistiocytosis (HLH) is a syndrome of known severe immune dysregulation characterized by the uncontrolled activation of macrophages and cytotoxic lymphocytes, leading to hyperinflammatory states and multi-organ dysfunction. HLH is often seen classified into two forms: primary (familial) HLH, which is caused by mutations in genes regulating T-cell and natural killer (NK) cell function (e.g., PRF1, UNC13D, STXBP2), and secondary HLH, which arises as a response to infections, autoimmune diseases, or malignancies (m-HLH). In pediatric patients, m-HLH is most commonly associated with hematologic malignancies, such as leukemia and lymphoma, but also occurs in solid tumors and post-hematopoietic stem cell transplantation.

The derived pathophysiology of HLH involves excessive activation of immune cells, particularly CD8+ T cells and macrophages, leading to the overproduction of pro-inflammatory cytokines, such as interferon-gamma (IFN-γ) interleukin-6 (IL-6) and tumor necrosis factor-alpha (TNF-α). This cytokine storm then ends up resulting in severe systemic inflammation, tissue damage; if untreated, multi-organ failure can also end up occurring. Despite its distinct pathogenesis, m-HLH frequently presents with symptoms that overlap with underlying malignancies, complicating early diagnosis and delaying appropriate treatment initiation.

In the context of m-HLH in pediatric patients, the initial presentation presents a diagnostic conundrum. The HLH-2004 diagnostic criteria include clinical features of fever, splenomegaly, and cytopenia, which overlap with common malignancies of leukemia and lymphoma. However, these criteria are not always fully applicable to m-HLH, particularly in cases where malignancy-related bone marrow suppression or even systemic inflammation may obscure diagnostic findings [1]. There are also known similarities in treatment guidelines with the use of dexamethasone and etoposide. Patients are known as critically ill at presentation, which prevent opportunities to carry out thorough diagnostic processes prior to treatment. This leads to delays in the diagnosis and treatment of underlying malignancy. The Histiocyte Society recommends evaluating all suspected HLH patients for malignancy, but there are currently no standardized guidelines for pediatric m-HLH regarding the following factors:

1. The initial diagnostic approach, including whether and when to perform bone marrow biopsy, flow cytometry, or genetic testing for HLH-related mutations.
2. The treatment focus: whether to prioritize HLH-directed immunosuppression or start malignancy-targeted chemotherapy first.
3. The modification of standard HLH treatment protocols in cases where chemotherapeutic toxicities may further compromise an already critically ill patient.

Treatment strategies for m-HLH typically follow HLH-94 or HLH-2004 protocols, including dexamethasone, etoposide, cyclosporine, and, in some cases, hematopoietic stem cell transplantation (HSCT) for refractory cases. However, these regimens were primarily developed for familial HLH and may not be optimal for m-HLH, where underlying malignancy requires treatment with the same time frame. The emergence of targeted immunotherapies, such as ruxolitinib (a JAK1/2 inhibitor) and emapalumab (an IFN-γ inhibitor), provides new options for refractory HLH. Nonetheless, their role in pediatric m-HLH remains under investigation, and there is limited guidance on integrating them into standard treatment algorithms [2].

Given these gaps, further research is needed to develop pediatric/adolescent-specific diagnostic and treatment protocols that balance the dual goals of HLH control and malignancy management [3]. Biomarker-driven risk stratification, and earlier use of targeted therapies, and consensus guidelines on the sequencing of HLH and malignancy-directed therapies could significantly improve outcomes.

**Figure 1:**
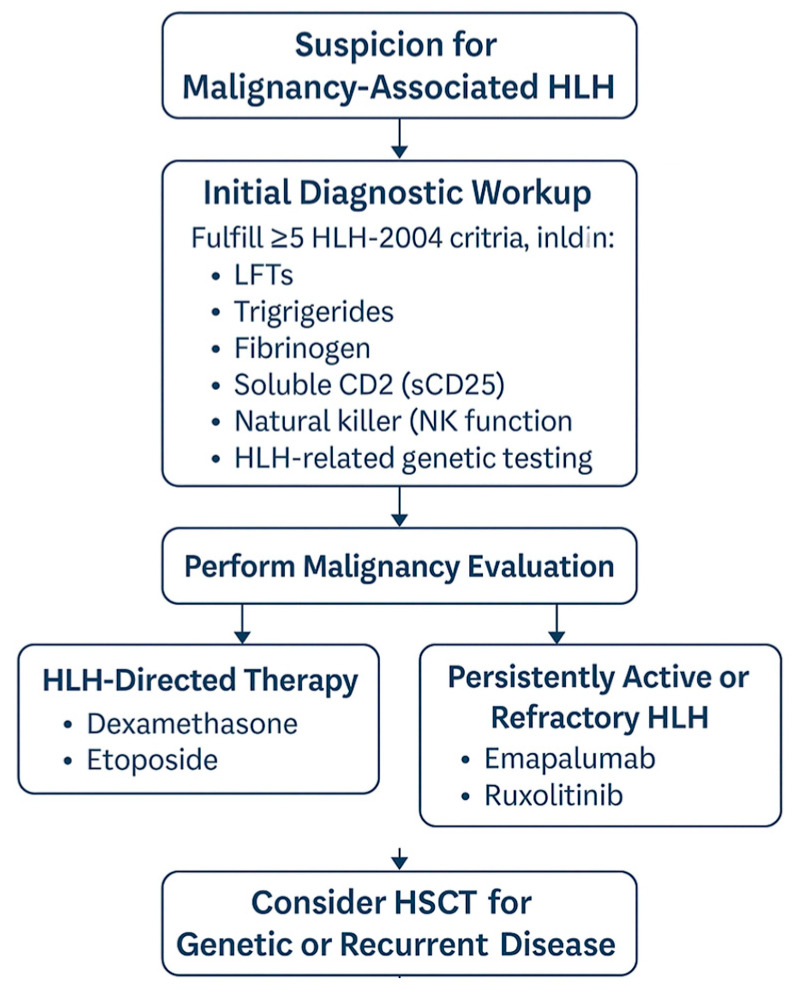
Diagnostic and treatment algorithm for pediatric malignancy-associated hemophagocytic lymphohistiocytosis (HLH). The pathway includes initial suspicion, diagnostic workup per HLH-2004 criteria, malignancy evaluation, and tailored therapy with escalation to HLH-directed agents or targeted therapies in refractory cases. Hematopoietic stem cell transplant (HSCT) is considered for patients with genetic or recurrent disease.

This case series highlights three adolescent patients with m-HLH, illustrating the complexity of diagnosis, treatment decisions, and response to novel therapies. Through these cases, we aim to provide insights into the evolving landscape of m-HLH management and underscore the urgent need for standardized guidelines. A proposed diagnostic and treatment algorithm for pediatric malignancy-associated HLH is presented in Figure 1, offering a structured approach to guide clinical decision-making [4].

## 2. Patient Cases

### 2.1. Patient Cases

#### 2.1.1. Patient 1 

A 19-year-old Hispanic male with a history of asthma presented with stage IIB high-risk classical Hodgkin’s lymphoma (HL), diagnosed and treated per Children’s Oncology Group (COG) protocol AHOD1331. His treatment regimen included brentuximab vedotin, doxorubicin, vincristine, etoposide, prednisone, and cyclophosphamide. His disease course was complicated by EBV-associated HLH, which presented during treatment [5]. The HLH manifested with splenomegaly, elevated ferritin levels exceeding 20,000 ng/mL, low NK cell activity, and high fevers.

The timing of the HLH diagnosis was during ongoing treatment for his Hodgkin’s lymphoma, not at initial diagnosis. Given his clinical presentation and the significant overlap of symptoms, including fevers and cytopenia, his HLH was suspected to be a treatment complication. At the time of HLH diagnosis, he was thoroughly evaluated for inciting infections, and tests for EBV reactivation and other infections were closely monitored to rule out infectious causes.

Initial HLH therapy included high-dose dexamethasone, etoposide, and anakinra. These treatments stabilized his inflammatory markers, but due to persistent inflammation, tachycardia, and insufficient control with conventional therapy, the patient was transitioned to ruxolitinib (a JAK1/2 inhibitor) and continued anakinra as an outpatient. After transitioning to this regimen, the patient showed significant clinical improvement, including the resolution of fevers, stabilization of heart rate, and normalization of ferritin levels to around 1600 ng/mL.

However, his treatment was complicated by severe neutropenia (ANC < 500/μL) and thrombocytopenia (platelet count < 50,000/μL), along with the ongoing risk of EBV reactivation, requiring close viral load monitoring. The HLH therapy-induced cytopenias did cause delays in some cycles of his Hodgkin’s lymphoma treatment, but his therapy was adjusted accordingly to maintain the balance between treating both conditions. Despite these challenges, the patient successfully completed chemotherapy for Hodgkin’s lymphoma and achieved complete remission.

His HLH remained well-controlled with outpatient ruxolitinib and anakinra, without the need for further etoposide therapy. The patient remains in remission for both Hodgkin’s lymphoma and HLH, and he is not currently receiving any therapy for HLH.

#### 2.1.2. Patient 2

A 16-year-old Hispanic female with persistent NK/T-cell lymphoma of the nasopharynx presented in septic shock with pancytopenia and disseminated intravascular coagulation (DIC). Initial diagnostic considerations included progressive lymphoma, bacteremia, and EBV viremia. HLH was considered, but she did not meet the criteria for diagnosis until hospital day 7. At that point, despite initiating therapy with dexamethasone, etoposide, and rituximab, the patient showed minimal clinical response, prompting further investigation into the cause of her HLH. There was increasing concern that her lymphoma itself might be driving the HLH.

A bone marrow biopsy confirmed the presence of NK/T-cell lymphoma, providing clarity on the underlying malignancy. In response to the worsening clinical picture, gemcitabine and oxaliplatin were initiated as lymphoma-directed therapy. However, the patient rapidly decompensated, experiencing hemodynamic instability and multi-organ system failure due to fulminant EBV viremia. Her clinical condition worsened as systemic inflammation escalated, reflected by significant rises in ferritin, soluble interleukin-2 receptor (sIL-2R), and CXCL9 (a biomarker for interferon-gamma). The elevated sIL-2R to ferritin ratio of 35:1 was notable and consistent with studies suggesting a strong correlation between lymphoma and HLH.

In light of her continued deterioration and increasing toxicity from lymphoma therapy, her lymphoma treatment was paused to reduce further toxic effects and to focus on more aggressive management of her HLH. Given the severity of the situation and the lack of improvement with conventional therapies, the decision was made to initiate emapalumab, an IFNγ inhibitor indicated in primary HLH. The administration of emapalumab resulted in a marked reduction in systemic inflammatory processes, with a resolution of fevers, significant decreases in inflammatory markers, and signs of recovery from hepatobiliary, renal, and gastrointestinal dysfunctions.

Despite the promising response to emapalumab, the patient’s condition continued to deteriorate. She developed fulminant candidiasis, which significantly worsened her clinical state, and further complications ensued with intraparenchymal hemorrhage. The patient’s multi-organ failure ultimately led to her death, despite the intervention of emapalumab and the temporary stabilization of inflammatory markers.

#### 2.1.3. Patient 3

A 17-year-old Caucasian male presented with pancytopenia and proctitis and was diagnosed with T-ALL and treated per COG-AALL1231 [6]. His course was complicated by bacteremia, fungemia, severe myelosuppression, and persistent fevers. While undergoing treatment for T-ALL, he met HLH criteria and was diagnosed with HLH after several weeks of chemotherapy [7]. His HLH was treated per HLH-2004 guidelines. Due to his myelosuppression, adjustments were made to both his chemotherapy and his HLH treatment. Reduced doses of dexamethasone were used, and etoposide was not introduced initially. Trials of IVIG and hydrocortisone showed minimal response.

We then trialed ruxolitinib, a JAK 1/2 inhibitor shown to improve the inflammatory status of patients with HLH [8]. Although laboratory markers did not indicate resolution of his HLH, while our patient was on ruxolitinib, he had an overall improved quality of life. He had more energy, improved wound healing, and significantly fewer admissions for flares for 3 months. Abdominal imaging demonstrated diffuse hepatic fungal microabscesses confirmed with biopsy.

He was previously treated with courses of voriconazole and micafungin, but doses were adjusted due to incompatibility with chemotherapy and concurrent toxicities. Treatment with a course of high dose fluconazole and repeat imaging showed resolution of the hepatic nodules. For his HLH, we trialed a course of etoposide, which was not used before due to his myelosuppression, but HLH did not improve. With our patient’s fungal infection controlled, we began a trial of emapalumab which lasted approximately three months and resulted in progressive improvement of his inflammation, which has allowed for progression of his treatment for his T-ALL. 

**Figure 2:**
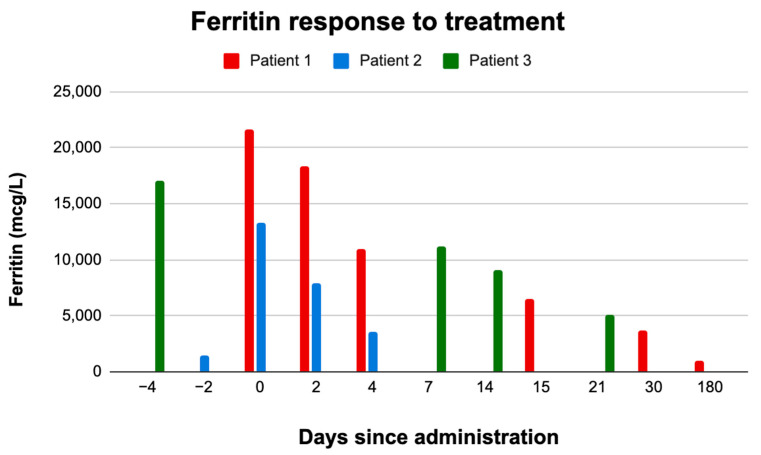
Ferritin levels over time in pediatric patients with refractory malignancy-associated HLH.

Currently, his HLH and T-ALL both remain in remission and he has completed maintenance therapy for his leukemia. As shown in Figure 2, ferritin levels significantly fluctuate during treatment in patients with refractory m-HLH, with novel therapies like ruxolitinib demonstrating substantial effects on ferritin normalization. Table 1 displays the changes in other lab work for key clinical and laboratory findings, providing insight into the response of pediatric m-HLH patients to various therapeutic interventions.

## 3. Discussion

These cases show the details of the management of m-HLH in pediatric patients within a spectrum of disease severity and complex diagnostic evaluations that one considers at presentation. Careful consideration must be taken with suspicion of HLH to carry out a thorough workup that will prevent delays in diagnosis for possible underlying malignancies. 

While our case series illustrates the complexity of diagnosing malignancy-associated HLH (m-HLH) in pediatric patients, it does not provide definitive diagnostic criteria or treatment guidance. The overlapping features of malignancy, chemotherapy side effects, and HLH create substantial ambiguity. This limitation underscores the need for more precise pediatric-specific definitions and biomarkers. NK cell functional testing was not performed due to limited availability and the acute clinical deterioration of the patients. This is a limitation in applying the full HLH-2004 diagnostic criteria.

Given this complex immunological and clinical environment, the use of dexamethasone and etoposide, which are standard treatments for HLH, may not be appropriate for all patients. While these medications are effective in managing the inflammatory response associated with HLH, they also carry significant risks in immunocompromised patients. Both dexamethasone and etoposide have marrow-suppressive effects, which can further reduce the already impaired bone marrow function in patients undergoing chemotherapy for malignancies like leukemia or lymphoma. This suppression not only increases the risk of infections but also heightens the potential for severe neutropenia, thrombocytopenia, and anemia, all of which can further complicate the clinical picture and delay recovery.

The cases of Patients 1 and 2 can be shown to illustrate the promise of ruxolitinib and emapalumab for refractory m-HLH. Patient 1 experienced significant relief with ruxolitinib, including reduced fever and stabilized ferritin levels, but ended up developing severe neutropenia and thrombocytopenia, requiring close monitoring for infections like EBV reactivation. This shows the risks of using immunosuppressive therapies in immunocompromised patients.

In Patient 2, emapalumab effectively reduced systemic inflammation and improved organ function, but the patient developed fulminant candidiasis and intraparenchymal hemorrhage, which she ultimately succumbed to. The adverse outcome may be due to emapalumab’s inhibition of IFN-γ, impairing neutrophil function and ultimately increasing susceptibility to fungal infections. This highlights a critical safety concern with immune-modulating therapies.

Toxicity management is crucial in pediatric m-HLH, particularly given the overlap between HLH treatments and chemotherapy. In Patient 3, managing both T-ALL and HLH required adjustments to treatment. Reduced dexamethasone doses while avoiding etoposide were necessary due to severe myelosuppression. Although it provided symptomatic relief, ruxolitinib also did not fully resolve HLH. After controlling a fungal infection, emapalumab led to progressive improvement, which enabled the continuation of leukemia treatment. This case highlights the potential of novel therapies like emapalumab when standard treatments are insufficient or poorly tolerated.

The treatment of m-HLH is a delicate balance due to the myriad of toxicities that arise from HLH and malignancy protocols. Although they are the backbone of HLH treatment, dexamethasone and etoposide may be inappropriate for some of the more unstable patients. This also highlights the importance of recognizing a lack of response to standard treatment to explore the use of investigational therapeutics for refractory cases in a timely manner. There is much to explore and learn from the use of novel therapeutics as with our patients who received emapalumab for their refractory HLH. Although they both demonstrated responses to the addition of emapalumab, one of our patients may have succumbed to fulminant candidiasis as result of emapalumab. We theorize that as emapalumab blocks IFNγ, subsequently blocking neutrophil activation and IL-6 modulation; it led to our patient being more susceptible to systemic candidiasis, resulting in fungal emboli that led to her intraparenchymal hemorrhage [8]). Both ruxolitinib and emapalumab show promising effects in patients but present safety concerns. In particular, the use of emapalumab, which blocks IFNγ, may lead to increased susceptibility to infections such as systemic candidiasis, potentially contributing to adverse outcomes [9].

Due to treatment for concurrent processes of malignancy, infection, and HLH, novel therapeutics such as emapalumab and ruxolitinib are often toxic yet required; they should be evaluated in larger studies. Considerations for the diagnostic approach of m-HLH should account for significant correlations like those seen in lymphoma-associated HLH and the sIL-2 to ferritin ratio [10]. The management of pediatric and adolescent m-HLH is a concurrently evolving field, and the experiences from these case reports underscore the complexities and challenges in diagnosis, treatment, and toxicity management [11,12]. While novel immunotherapies such as ruxolitinib and emapalumab show promise, their role in pediatric m-HLH remains under investigation to this day and therefore, safety concerns, particularly related to infections, must be carefully considered and taken account of. There is an urgent need for standardized guidelines that incorporate both traditional and novel treatments, as well as a more individualized approach to therapy based on disease severity, underlying malignancy, and patient-specific factors [13].

## Figures and Tables

**Table 1:** Post-administration laboratory and clinical findings in pediatric patients with refractory malignancy-associated HLH.

	Patient 1						Patient 2 **				Patient 3				
Days since administration	0	2	4	15	30	180	−2	0	2	4	−4	0	7	14	21
Tmax	36.1	36.2	36.5	36.4	36.4	36.4	39.3	37.4	36.7	38.4	36.7	36.4	36.4	36.4	36.3
wbc	33.2	25	12.4	1.5	21.2	4.9	0.1	0.1	0.1	0.1	1.5	0.9	1.6	2.9	1.5
Hgb	7.5	7.1	8.9	8.6	10.6	13.2	7.3	7.9	8.3	5.4	7.3	8.1	7.5	7.6	9.1
Hct	23.4	21.1	26.4	25.3	32	38.8	20.5	23.2	23.3	15.4	21.7	24	22.6	22.5	25.9
Mcv	84.3	84.1	86.3	86.3	94.9	101.7	80.1	81.7	79.8	78.4	97.1	93.5	96.2	98.9	99.7
Plt	833	524	391	242	449	215	13	19	44	25	25	25	21	33	29
Ferritin	21,627	18,359	10,949	6470	3714	1046	1459	13,279	7889	3604	17,094	*	11,139	9081	5153
AST	63	50	84	60	*	40	618	421	251	68	220	204	155	133	77
ALT	89	80	178	190	*	45	240	185	137	37	427	364	259	213	168
TG	*	*	*	*	*	*	595	1188	1890	814	476	*	*	*	*
Fibrinogen	*	*	*	*	*	*	101	135	155	194	430	*	349	430	438

HLH = hemophagocytic lymphohistiocytosis; ANC = absolute neutrophil count; WBC = white blood cell count; AST = aspartate aminotransferase; ALT = alanine aminotransferase; MCV = mean corpuscular volume; DIC = disseminated intravascular coagulation. * Value not obtained. ** Patient expired.

## Data Availability

The data that support the findings of this study are available from the corresponding author upon reasonable request.
